# Pleiotropic antifibrotic actions of aspirin-triggered resolvin D1 in the lungs

**DOI:** 10.3389/fimmu.2023.886601

**Published:** 2023-03-07

**Authors:** Rafael F. Guilherme, José Bruno N.F. Silva, Ingrid Waclawiack, Vanderlei S. Fraga-Junior, Thaís O. Nogueira, Cyntia Pecli, Carlla A. Araújo-Silva, Nathalia S. Magalhães, Felipe S. Lemos, Carlos A. Bulant, Pablo J. Blanco, Rafaela Serra, Erik Svensjö, Júlio Scharfstein, João A. Moraes, Claudio Canetti, Claudia F. Benjamim

**Affiliations:** ^1^ Instituto de Microbiologia Paulo de Góes, Universidade Federal do Rio de Janeiro, Rio de Janeiro, Brazil; ^2^ Laboratório de Biotecnologia, Imunobiologia e Estudos em Saúde, Universidade Federal do Tocantins, Palmas, TO, Brazil; ^3^ Instituto de Biofísica Carlos Chagas Filho, Universidade Federal do Rio de Janeiro, Rio de Janeiro, Brazil; ^4^ Instituto de Ciências Biomédicas, Universidade Federal do Rio de Janeiro, Rio de Janeiro, Brazil; ^5^ Laboratório de Pesquisa em Infecção Hospitalar, Instituto Oswaldo Cruz, Fiocruz, Rio de Janeiro, Brazil; ^6^ Laboratório de Imunofarmacologia, Instituto Oswaldo Cruz, Fiocruz, Rio de Janeiro, Brazil; ^7^ Pladema Institute, National Scientific and Technical Research Council (CONICET), Tandil, Buenos Aires, Argentina; ^8^ Departamento de Métodos Matemático e Computacional, Laboratório Nacional para Computação Científica, Rio de Janeiro, Brazil

**Keywords:** ATRvD1, lung, inflammation, fibrosis, macrophages, angiogenesis, microparticles, tissue repair

## Abstract

**Introduction:**

Pulmonary fibrosis is a destructive, progressive disease that dramatically reduces life quality of patients, ultimately leading to death. Therapeutic regimens for pulmonary fibrosis have shown limited benefits, hence justifying the efforts to evaluate the outcome of alternative treatments.

**Methods:**

Using a mouse model of bleomycin (BLM)-induced lung fibrosis, in the current work we asked whether treatment with pro-resolution molecules, such as pro-resolving lipid mediators (SPMs) could ameliorate pulmonary fibrosis. To this end, we injected aspirin-triggered resolvin D1 (7S,8R,17R-trihydroxy-4Z,9E,11E,13Z,15E19Z-docosahexaenoic acid; ATRvD1; i.v.) 7 and 10 days after BLM (intratracheal) challenge and samples were two weeks later.

**Results and discussion:**

Assessment of outcome in the lung tissues revealed that ATRvD1 partially restored lung architecture, reduced leukocyte infiltration, and inhibited formation of interstitial edema. In addition, lung tissues from BLM-induced mice treated with ATRvD1 displayed reduced levels of TNF-α, MCP-1, IL-1-β, and TGF-β. Of further interest, ATRvD1 decreased lung tissue expression of MMP-9, without affecting TIMP-1. Highlighting the beneficial effects of ATRvD1, we found reduced deposition of collagen and fibronectin in the lung tissues. Congruent with the anti-fibrotic effects that ATRvD1 exerted in lung tissues, α-SMA expression was decreased, suggesting that myofibroblast differentiation was inhibited by ATRvD1. Turning to culture systems, we next showed that ATRvD1 impaired TGF-β-induced fibroblast differentiation into myofibroblast. After showing that ATRvD1 hampered extracellular vesicles (EVs) release in the supernatants from TGF-β-stimulated cultures of mouse macrophages, we verified that ATRvD1 also inhibited the release of EVs in the bronco-alveolar lavage (BAL) fluid of BLM-induced mice. Motivated by studies showing that BLM-induced lung fibrosis is linked to angiogenesis, we asked whether ATRvD1 could blunt BLM-induced angiogenesis in the hamster cheek pouch model (HCP). Indeed, our intravital microscopy studies confirmed that ATRvD1 abrogates BLM-induced angiogenesis. Collectively, our findings suggest that treatment of pulmonary fibrosis patients with ATRvD1 deserves to be explored as a therapeutic option in the clinical setting.

## Introduction

Pulmonary fibrosis is a severe disease of unknown cause with a median fatal outcome of 3 years after diagnosis (1, 2). Characterized by interstitial inflammation and scarring around the alveoli, the progressive formation of scars is thought to reflect excessive synthesis and deposition of extracellular matrix, a pathological feature ascribed to the upregulated proliferation of fibroblasts and differentiation into myofibroblasts. In order to prevent dyspnea and progressive organ dysfunction (3–5), attempts have been made to curb the escalation of the fibrotic process mainly through the administration of glucocorticoids alone or in association with immunosuppressive agents (6), of anti-fibrotic agents such as pirfenidone (7), and of a tyrosine kinase inhibitor such as nintedanib (8). Despite the relief of clinical symptoms, these therapeutic approaches did not translate into the long-term survival of pulmonary fibrosis patients (9). Out of other therapeutic options, treatment of patients with pulmonary fibrosis depends on lung transplantation, an invasive procedure that has a high risk of failure (10). A major challenge in this field is to develop alternative strategies for pharmacological contention of the progression of interstitial fibrosis, a pathological process that is commonly observed in other lung-associated diseases (11).

Among the possible candidates displaying pro-resolution activities, aspirin-triggered resolvin D1 (7S,8R,17R-trihydroxy-4Z,9E,11E,13Z,15E19Z-docosahexaenoic acid; ATRvD1) is particularly interesting because it belongs to the group of specialized pro-resolving lipid mediators (SPMs) generated from the omega-3 fatty acid docosahexaenoic acid, by aspirin-acetylated cyclooxygenase-2 (12). The biological effects of ATRvD1 depend on the ligand-mediated signaling of both lipoxin receptor (ALX)/N-formyl peptide receptor (FPR)-2 (ALX/FPR2) and GPR32 (12), members of the G protein-coupled receptor superfamily. Cellular signaling by ATRvD1 is involved in a myriad of biological responses, including inhibition of hyperalgesia (13), protection from hydrochloric acid- and hyperoxic-induced acute lung injury (14, 15), modulation of allergic airway responsiveness (16), regulation of fibrosis induced by mechanical stretch (17), endotoxin-induced acute kidney injury (18), and ischemia/reperfusion injury in the liver (19).

Using a mouse model of pulmonary fibrosis induced by bleomycin (BLM), Iyer and colleagues demonstrated that nitric oxide mediates BLM-induced angiogenesis and pulmonary fibrosis *via* upregulation of vascular endothelial growth factor (VEGF) (20). Yatomi and colleagues provided the first precedent that ATRvD1 ameliorated BLM-induced pulmonary fibrosis in mice by decreasing collagen deposition and cellular accumulation and restoring the matrix metallopeptidase 9 (MMP-9) expression (21). Beyond confirming their original findings, here we provide new antifibrotic mechanisms of ATRvD1 using *in-vivo* and *in-vitro* experimental models such as BLM-induced fibrosis in mice, murine fibroblasts culture, and hamster cheek pouch (HCP). Our results suggest that antifibrotic ATRvD1 effects are due to pleiotropic actions, including inhibition of the release of inflammatory mediators, cellular accumulation, fibroblast differentiation, extracellular vesicle (EV) production, and angiogenesis inhibition.

## Material and methods

### Animals

Male and female C57BL/6 mice (8-12 weeks) weighing 18-22 g were obtained from the Brazilian National Institute of Cancer (INCA, Rio de Janeiro, Brazil) and Multidisciplinary Center for Biological Investigation (University of Campinas, São Paulo, Brazil). Three-month-old male Syrian hamsters, weighing 110 to 120 g, were purchased from Anilab (São Paulo, Brazil) and maintained in our animal facility according to regulations given by the local ethics committee (CEUA UFRJ: 021/16 and 158/18). Animals were housed at constant temperature of 25°C under a 12-h light/dark cycle with free access to food and water. All procedures were performed according to the guidelines of the Committee on Ethical Use of Laboratory Animals of the Federal University of Rio de Janeiro (CEUA UFRJ: 092/14).

### The fibrosis model and ATRvD1 treatment

The pulmonary fibrosis model was induced by intratracheal instillation (i.t.) of BLM [Sigma Aldrich, Saint Louis, MO, USA; 0.06 U/mouse in a final volume of 30 μl of saline (SAL)] as previously described (22, 23). Mice were divided into three groups: 1) SAL group = mice received i.t. administration of SAL and i.v. administration of ethanol 0.1% and 0.01% (diluted in SAL) on days 7 and 10, respectively; 2) BLM group = mice received BLM i.t. injection on day 0 and i.v. administration of SAL on days 7 and 10, respectively; and 3) BLM + ATRvD1 group = mice received BLM i.t. injection on day 0 and i.v. administration of ATRvD1 (Cayman Chemicals, Ann Arbor, MI, USA) 2.5 μg/kg in 0.1% ethanol and 0.5 μg/kg in 0.02% ethanol on days 7 and 10, respectively. On day 14, bronchoalveolar lavage (BAL) was performed. The lungs were immediately removed postmortem and used for histological analysis, cytokine quantification by ELISA, and detection of primary cell-derived EVs.

### Histological analysis

The tracheas were clamped at the end-expiration, the lungs were removed *en bloc* and immersed in buffered formalin (10%) for 48 h before embedding in paraffin, and sections (5 μm thick) were obtained. Histologic sections were stained with hematoxylin and eosin for structural analysis or by Picrosirius red staining to evaluate matrix protein deposition, mainly collagen. The tissue sections stained with Picrosirius red were also analyzed by polarized light microscopy. Areas were photographed with an Olympus BX53 light microscope (Tokyo, Japan).

### Immunohistochemistry and immunofluorescence assays

Lung sections were immunostained with antibodies against collagen type I (Santa Cruz Biotechnology, Santa Cruz, CA, USA) and alpha-smooth muscle actin (α-SMA; Abcam, Cambridge, MA, USA). Immunohistochemistry was performed as previously described (24).

For immunofluorescence, lung sections were immunolabeled with primary antibodies against α-SMA and fibronectin (Abcam, Cambridge, MA, USA) at 1:400 and 1:800 dilutions, respectively. The slides were incubated with fluorescent secondary goat anti-rabbit IgG H&L (Alexa Fluor^®^ 488; Invitrogen, Carlsbad, CA, USA) diluted 1:400 in blocking solution. The slides were counterstained with VectaShield with DAPI (Vector Laboratories, Burlingame, CA, USA) for nuclei staining.

### BAL

BAL was performed as described elsewhere (25). Briefly, BAL was performed by the i.t. administration of phosphate-buffered saline (PBS) solution (1 ml), and after a thoracic massage, the instilled fluid was gently retracted to maximize BAL fluid recovery and to minimize shearing forces.

### Leukocyte analysis

We performed total and differential counts in the BAL fluid. The cells were diluted in Turk’s solution (1:10), and the total leukocyte count was performed in a Neubauer chamber. Mononuclear cells and neutrophils were quantified on Diff-Quik-stained cytocentrifuge slides (Cytospin 3; Shandon, Thermo Fisher Sientific, Waltham, MA). The data were expressed as the number of cells (×10^5^) per ml.

### Hydroxyproline assay

In order to determine fibrosis, the right lung tissue was prepared for hydroxyproline assay as previously described by Woessner (26). Lung hydroxyproline content was determined spectrophotometrically by absorbance at 550 nm, and the results were expressed as nanograms of hydroxyproline per milligram of tissue.

### ELISA

Lung samples were prepared from the whole lung homogenized in 1 ml of PBS containing a mixture of protease inhibitors (Complete; Sigma-Aldrich, Switzerland). The levels of interleukin (IL)-1β, MMP-9, monocyte chemoattractant protein-1 (MCP-1/CCL-2), tissue inhibitor of metalloproteinases 1 (TIMP-1), TGF-β, and tumor necrosis factor-alpha (TNF-α) in the lung samples were measured by ELISA (R&D Systems, Minneapolis, MN, USA), according to the manufacturer’s instructions. The results were expressed as ng/mg of protein (27).

### Fibroblast and macrophage culture

Lung tissues were cut into small pieces of 1 mm^3^ and then incubated in collagenase I 0.2% (Sigma-Aldrich) for 1.5 h at 37°C. After the incubation, the samples were centrifuged, and the pellet was incubated with ammonium chloride potassium bicarbonate (ACK) lysing buffer for blood cell depletion. After centrifugation, cells were resuspended in DMEM F12 medium, supplemented with 10% fetal calf serum (FCS) and 100 U/ml of penicillin/streptomycin. The cell suspension was subsequently transferred to a flask and placed at 37°C in a 5% CO_2_ incubator, and the experiments were performed from the third to the eighth passages. Primary fibroblasts (1 × 10^5^ cells/ml) were plated with DMEM F12 medium (unstimulated) and incubated with TGF-β (10 ng/ml) or with TGF-β + ATRvD1 (100 ng/ml) for 24 h.

Alveolar macrophages were collected *via* BAL. After cell count, the cells were resuspended in DMEM F12 medium with 10% FCS and adhered for 2 h. Thus, cultures were washed and incubated for further 18 h to reduce the presence of B1 cells. Macrophages were cultured in medium alone (unstimulated) or with TGF-β (10 ng/ml) or with TGF-β + ATRvD1 (100 ng/ml) for 24 h. Culture purity was assessed by flow cytometry with 95% of macrophage cells (F4/80^+^ cells). The supernatants obtained were subjected to EV analysis by flow cytometry.

### Isolation and quantification of EVs

Plasma or conditioned media from macrophages were collected and centrifuged at 250×*g* for 10 min, and the supernatants were stored at −20°C. To obtain the EVs, the supernatant from plasma or conditioned media were (ultra)centrifuged at 100,000×*g* for 4 h. The pellets were resuspended in annexin V buffer or FACS buffer and were stored at −20°C until use. The EVs were assessed using an Accuri™ C6 Flow Cytometer (Becton Dickinson, Franklin Lakes, NJ, USA) through the evaluation of annexin V (Thermo Fisher Scientific, Waltham, MA, USA), F4/80-PE (Biolegend, San Diego, CA, USA), SinglecF-alexa488, LY6G-APC, and CD3-alexa488 (BD Pharmingen, San Diego, CA, USA)-positive events. The gated region containing the EVs was previously delimited by using known concentrations of 1-µm beads to estimate the precise amount of EVs, as previously described (28).

### Flow cytometry analysis

The lungs were collected on day 14 after SAL or BLM installation. Briefly, the lungs obtained from each mouse were digested by collagenase A (1 mg/ml; Sigma-Aldrich) for at least 40 min, with shaking at 37°C. Phosphate-buffered saline containing 10% fetal bovine serum was added to inactivate the enzyme, and the solution was passed through a 40-µm cell strainer. Cells (10^6^ cells/ml) obtained from the lungs or BAL were incubated with LIVE/DEAD BV510 according to the manufacturer’s instruction (Life Technologies, Carlsbad, CA, USA) followed by anti-mouse CD16/32 Fc block (eBioscience, San Diego, CA) for 15 min at 4°C. Different cell populations were identified using the following corresponding antibodies: a) Ly6C-FITC, Ly6G-Alexa647, CD11b-Pecy7, CD11c-PECy5, and SinglecF-PE (monocytes, neutrophils, eosinophils, and alveolar macrophages) or b) CD11b-APC, CD11c-PECy5, SinglecF-FITC, CD86 PECy7, and CD206-PE (alveolar macrophage activation). Samples were acquired by BD LSRFortessa X-20 Cell Analyzer (BD Biosciences, San Rose, CA) and then analyzed by FlowJo software (Ashland, OR, USA).

### Total RNA extraction and quantitative real-time RT-PCR

Total RNA was extracted from lung tissue or 10^6^ primary mouse lung fibroblasts with the TRIzol reagent (Sigma) following the manufacturer’s procedures. Two micrograms of RNA was used for cDNA synthesis using the High-Capacity cDNA Reverse Transcription Kit (Life Technologies, Carlsbad, CA, USA). Quantitative real-time RT-qPCR was performed with the SYBR-green fluorescence quantification system. The PCR cycling parameters were 95°C (10 min) and then 40 cycles of 95°C (30 s) and 60°C (1 min), followed by the standard denaturation curve. The primer sets were as follows: GAPDH forward (F): 5′AGGTCGGTGTGAACGGATTTG; GAPDH reverse (R): 5′TGTAGACCATGTAGTTGAGGTCA; ALX (F): 5′GCCTTTTGGCTGGTTCCTGTGT; ALX (R): 5′CAAATGCAGCGGTCCAAGGCAA. ALX to GAPDH relative expression was calculated using the comparative Ct method.

### Intravital microscopy—microvascular measurements

Animals were anesthetized with an i.p. injection of ketamine (200 mg/kg) and xylazine (10 mg/kg) (IBCCF, protocol: 021/16 and 158/18). Then, using a cotton swab, the hamster cheek pouch (HCP; left) was inverted and mounted for intravital microscopy observation (without dissection) in an appropriate plate and topically treated with BLM (1 U/100 μl/hamster) with a sterile syringe (26½ G needle). The contralateral HCP received the same volume of SAL. Alternatively, 3 h post-BLM injection, some animals were treated with ATRvD1 (25 µg/kg - 100 µl i.v.) or SAL. After 24 h of BLM application, the animals were once again anesthetized and supplemented with i.v. α-chloralose (2.5% solution in SAL) through a femoral vein catheter. A tracheal cannula (PE 190) was inserted to facilitate spontaneous breathing, and the body temperature was maintained at 37°C by a heating pad monitored with a rectal thermometer. The HCP was prepared and used for intravital microscopy as described (29, 30). Then, the microcirculation of the HCP was assessed after i.v. injection of macromolecular tracer FITC-dextran 150 kDa (100 mg/kg; TdB Consultancy, Uppsala, Sweden) using an Axioskop 40 microscope, objective ×4, and oculars ×10, equipped with appropriate filters (490/520 nm) and a Colibri 2 LED-light source (Carl Zeiss, Germany). A digital camera, AxioCamHRc, and a computer with the AxioVision 4.4 software program (Carl Zeiss) were used for image recording and the subsequent measurement of fluorescence (relative fluorescent units, RFU). The methods for automatic characterization of the HCP microvasculature from intravital microscopy images were published by Bulant and colleagues (31). The image resolution is 1,388 × 1,040 for an area of approximately 5 mm^2^, which yields a pixel spacing of 1.862 µm and, therefore, a pixel area of 3.467 µm². At the end of each experiment, the animals were euthanized by an overdose of ketamine and xylazine or an i.v. injection of KCl 3 M.

### Statistical analysis

Differences between groups were analyzed by one-way ANOVA, followed by the Bonferroni *post-hoc* test. For all analyses, data were expressed as mean ± SEM. The GraphPad Prism 5 statistical software package (GraphPad Software, La Jolla, CA, USA) was used. A *P*-value <0.05 was considered significant.

## Results

### ATRvD1 impairs BLM-induced lung inflammation and fibrosis

**Figure 1:**
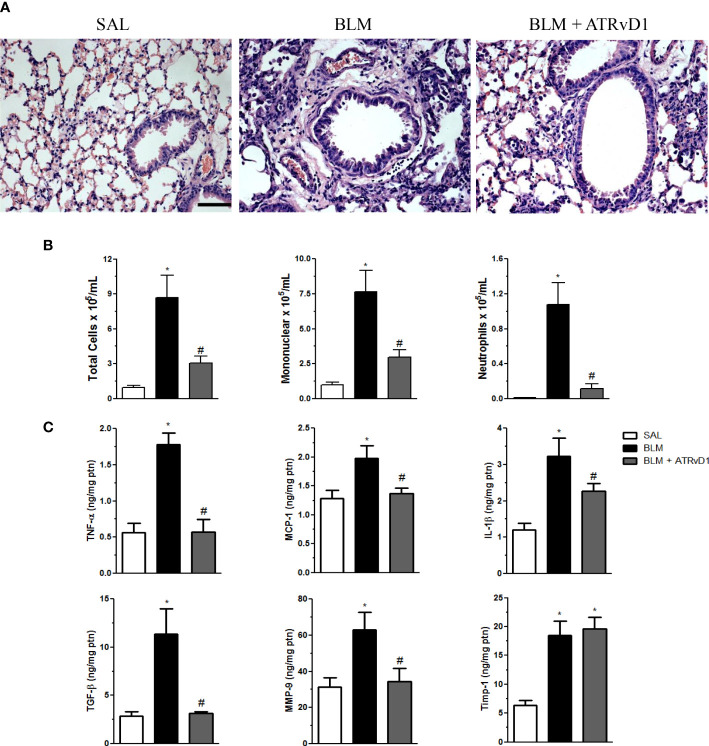
ATRvD1 treatment restores bleomycin (BLM)-induced lung morphology and inflammation. **(A)** Representative lung sections from saline (SAL) (left), BLM (center; 0.06 U/mouse in a final volume of 30 μl of SAL), and BLM plus ATRvD1 treatment (right; on day 7, 2.5 μg/kg and a booster on day 10 with 0.5 μg/kg) obtained 14 days after BLM injection and stained with hematoxylin and eosin. Scale bar = 50 μm. **(B, C)** Inflammatory cells and cytokines, MMP-9, and TIMP-1 were quantified in the BAL fluid and lung tissue, respectively, 14 days after SAL, BLM, or BLM plus ATRvD1 treatment as described in **(A)**. Results represent mean + SEM from two independent experiments. *P ≤ 0.05 vs. the SAL group; ^#^ *P* ≤ 0.05 vs. the BLM group.

In order to evaluate the effect of ATRvD1 treatment on BLM-established inflammation/fibrosis, mice were treated on days 7 and 10 after BLM (0.06 U/mouse) with ATRvD1 (25 and 5 μg/kg, respectively; i.v.) and sacrificed on the 14^th^ day, and the lung architecture was analyzed by histology. As observed in **Figure 1A**, an intense reaction occurred after the BLM challenge (middle panel), characterized by a high degree of inflammation, with cellular accumulation mainly in the interstitium, thickening and edema of the alveolar septa, and a marked reduction in open alveoli numbers compared with SAL-injected animals (left panel). Remarkably, ATRvD1 administrations after BLM resulted in lung structure re-establishment. Despite the tissue restructuration caused by ATRvD1 treatment, it is important to note that under the treatment approach adopted, we still can observe moderate signals of injury and inflammation, with the presence of inflammatory cells in the parenchyma and septa edema when compared with the SAL group (right panel).

Having determined that ATRvD1 treatment protects lung structures, we evaluated its effect on the cellularity, cytokine production, and MMP-9/TIMP-1 release in lung tissue evoked by BLM administration. The administration of BLM induced an increase in total cell recovery from the BAL fluid (**Figure 1B**, left graph), most of them being mononuclear cells (∼7.5 × 10^5^/ml, approximately 88% of recovered cells; middle graph) and minor extension neutrophils (∼1.1 × 10^5^/ml, approximately 11%, right graph). Interestingly, ATRvD1 treatment significantly decreased cell recruitment induced by BLM, as observed for total cells, mononuclear cells, and neutrophils (**Figure 1B**). Next, we observed that BLM-induced TNF-α, MCP-1/CCL-2, IL-1β, and TGF-β protein release was inhibited by ATRvD1 administration (**Figure 1C**). Additionally, ATRvD1 treatment was also able to impair BLM-induced MMP-9 expression, without affecting TIMP-1 expression (**Figure 1C**).

**Figure 2:**
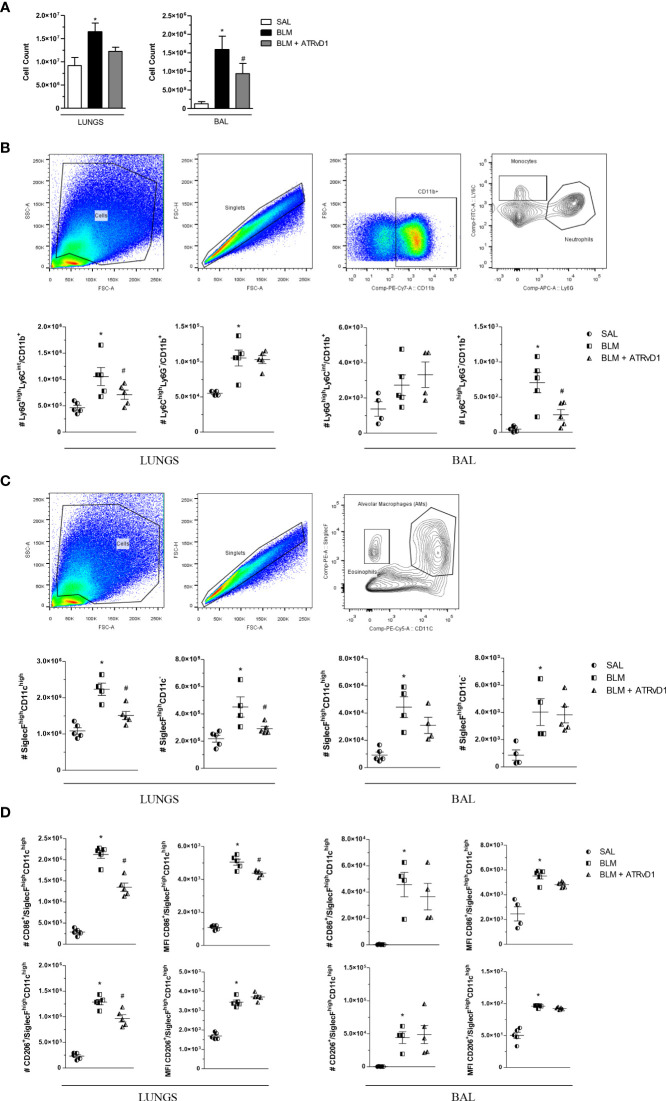
ATRvD1 rescues the cell numbers and phenotype in the lung and BAL of BLM-injected mice. **(A)** Total cell count quantified in the supernatant after enzymatic digestion of the lungs (left) and after BAL harvest (right). **(B)** Dot-plot images show the gate strategy for monocyte and neutrophil analysis: **(i)** forward and side scatter plots show a gated region representing leukocytes; **(ii)** forward scatter gated region of singlet cells; **(iii)** forward scatter and CD11b cell expression gated on live cells; **(iv)** contour plot of Ly6C^+^ and Ly6G^+^ cells. The bottom graphs show the absolute number of the neutrophil (Ly6G^high^Ly6C^int^/CD11b^+^) and monocyte (Ly6C^high^Ly6G^−^/CD11b^+^) populations in the lungs and BAL. **(C)** Dot-plot images show the gate strategy for alveolar macrophages and eosinophils: **(i)** forward and side scatter plots show a gated region representing leukocytes; **(ii)** forward scatter gated region of singlet cells; **(iii)** contour plot of CD11c and SiglecF gated on live cells. The bottom graphs show the absolute number of the alveolar macrophage (SiglecF^high^CD11c^high^) and eosinophil (SiglecF^high^CD11c^−^) populations in the lungs and BAL. **(D)** Absolute number and MFI of CD86^+^ or CD206^+^ cells in the SiglecF^high^CD11c^high^ population. Results represent mean + SEM from two independent experiments. *P ≤ 0.05 vs. the SAL group; ^#^ *P* ≤ 0.05 vs. the BLM group.

Using flow cytometry analysis, we confirmed the results obtained by the cell counter, in which BLM administration induced an increase in total cells, and ATRvD1 treatment significantly decreased BLM-induced cell recruitment into the lungs and BAL fluids (**Figure 2A**). Moreover, we observed an increase in Ly6C^high^Ly6G^−^/CD11b^+^ monocytes (**Figure 2B**), SiglecF^high^CD11c^−^ eosinophils, and SiglecF^high^CD11c^high^ alveolar macrophages (**Figure 2C**) in the lungs and BAL fluids and an increase in Ly6G^high^Ly6C^int^/CD11b^+^ neutrophils (**Figure 2B**) in the lungs of BLM-challenged mice. ATRvD1 treatment significantly decreased BLM-induced neutrophil, eosinophil, and alveolar macrophage accumulation in the lungs, while monocyte and alveolar macrophage recruitment was reduced in BAL fluids (**Figures 2B, C**). In order to investigate if ATRvD1 could interfere with macrophage polarization, we analyzed CD86 (M1 marker) and CD206 (M2 marker) expressions. We observed that the BLM challenge caused an increase in the number and MFI of CD86^+^ and CD206^+^ cells in the lungs and BAL fluids. ATRvD1 treatment decreased the number and MFI of CD86^+^ and the number of CD206^+^ macrophages in the lungs, without affecting their expression on alveolar macrophages (BAL) (**Figure 2D**).

### ATRvD1 treatment rescues extracellular matrix protein deposition induced by BLM

**Figure 3:**
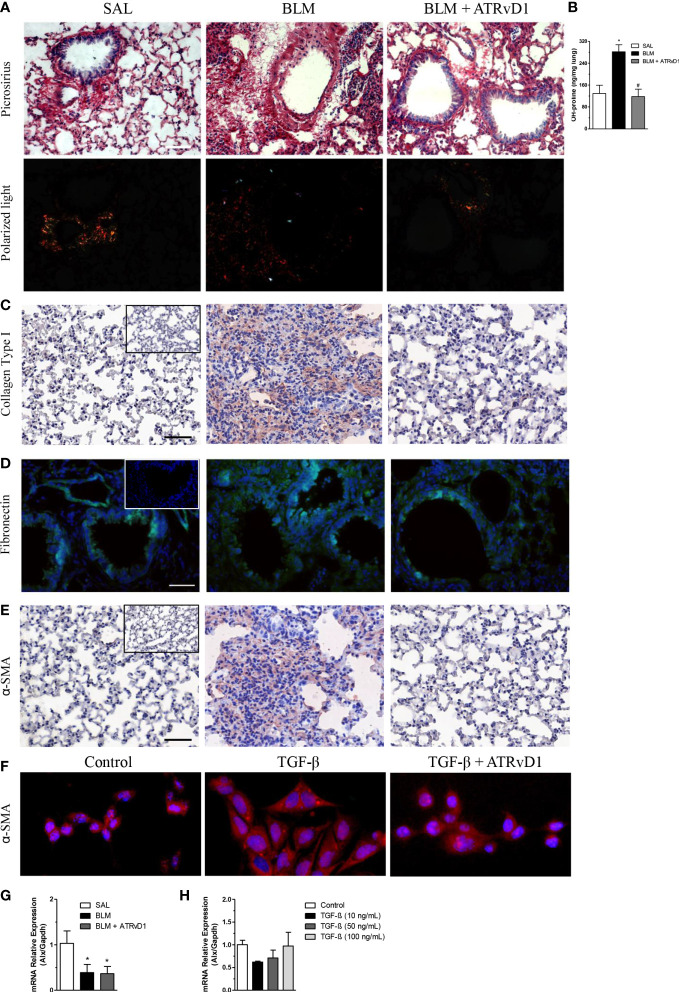
ATRvD1 impairs BLM-induced matrix protein deposition in the lung. Lungs were harvested on the 14th day after the SAL, BLM, or BLM + ATRvD1 challenges, as described in the *Material and methods* section. **(A)** Picrosirius red staining under bright field (superior images) and polarized light (lower images). **(B)** Hydroxyproline content in the lungs on the 14th day after mice were instilled with SAL, BLM, or BLM + ATRvD1 (top right). Results are expressed as micrograms per milligram of lung tissue. *P ≤ 0.05 compared with the SAL group; ^#^ *P* ≤ 0.05 compared with the BLM group. **(C–E)** Lung sections were immunostained for collagen type I and α-SMA by immunohistochemistry and counterstained with hematoxylin or for fibronectin and DAPI by immunofluorescence. **(F)** Primary lung fibroblasts were isolated, cultured, and incubated with the medium, TGF-β (10 ng/ml), or TGF-β + ATRvD1 (100 ng/ml) and then stained by immunofluorescence for α-SMA as described. Pictures are representative of each group, constituted of *n* = 4-5. Inserts in panels **(C–E)** represent the images obtained by immunostaining for the control isotype antibodies. **(G, H)** Total RNA was extracted from the lung tissue obtained from the SAL-, BLM-, or BLM + ATRvD1-treated mice or from the homogenate of 10^6^ primary mouse lung fibroblasts incubated with medium or TGF-β at 10, 50, and 100 ng/ml. Samples were prepared using the TRIzol reagent and quantitative real-time RT-qPCR for ALX expression determined as described in the *Material and methods* section. Scale bar = 50 μm.

The impact of ATRvD1 administration on BLM-induced extracellular protein deposition was evaluated by different strategies. In **Figure 3**, lung sections were stained by Picrosirius red, a technique commonly used to evaluate the presence of collagen fibers. As observed by ordinary light microscopy (panel A, upper line) and with polarized light (panel A, bottom line), BLM injection induced matrix protein deposition compared with the control group (SAL-injected mice), and ATRvD1 treatment reduced BLM-induced lung matrix protein deposition to a similar condition observed in the vehicle-injected mice. Substantially, we observed that ATRvD1 treatment also inhibited BLM-induced collagen deposition in lung tissue homogenates, indirectly evaluated by hydroxyproline contents (**Figure 3B**). Once again, the hydroxyproline amount observed in the ATRvD1 group was similar to those observed in the control mice. Furthermore, the protective effect of ATRvD1 in impairing BLM-induced matrix protein deposition was also confirmed by specific immune staining for collagen I. As observed in **Figure 3C**, collagen I staining in the lung of SAL-injected mice was limited to peribronchiolar areas; meanwhile, in BLM-treated mice, collagen I staining was spread over large areas of the parenchyma. Interestingly, ATRvD1 treatment was able to reduce collagen I deposition induced by the BLM challenge (**Figure 3C**). Regarding the effect of ATRvD1 on BLM-induced matrix protein deposition, we expanded our investigation to evaluate fibronectin contents in *in-vivo* experiments. As illustrated in **Figure 3D**, the BLM challenge induced the expression of fibronectin in the lung tissue compared with the control group, and similar to what occurred to the collagen contents, fibronectin expression was inhibited by ATRvD1 treatment, showing a similar intensity to those observed in the control group. In addition to our analyses on the 14th day, interestingly, on day 7 after BLM, when mice received the first administration of ATRvD1, it was already possible to detect cell recruitment, collagen deposit, and structural tissue damage in the lungs (**Supplementary Figure 1**).

Furthermore, we tested the effect of ATRvD1 treatment on BLM-induced α-SMA expression, an index of fibroblast differentiation into myofibroblasts. Immunohistochemistry analysis of the lung sections revealed that BLM caused an increase in α-SMA expression compared with the SAL-injected mice, which was reversed by ATRvD1 administration (**Figure 3E**). Next, to confirm the ATRvD1 effects on fibroblasts, we cultured primary isolated lung fibroblasts with and without TGF-β and evaluated the *in-vitro* differentiation *via* α-SMA expression. As demonstrated in **Figure 3F**, TGF-β-stimulated fibroblasts presented an increase in α-SMA expression compared with non-stimulated cells, whereas ATRvD1 co-incubation impaired myofibroblast differentiation.

Next, we investigated the ALX receptor mRNA expression in the lungs harvested from the control, BLM, and BLM-injected mice treated with ATRvD1. We detected the message for ALX in all groups; however, the mRNA expression was reduced in the lungs obtained from the BLM and ATRvD1-treated BLM samples (**Figure 3G**). Moreover, the incubation of fibroblasts with different TGF-β concentrations (10, 50, and 100 ng/ml) did not modify ALX mRNA expression (**Figure 3H**).

### ATRvD1 impairs EV formation *in vivo* and *in vitro*

**Figure 4:**
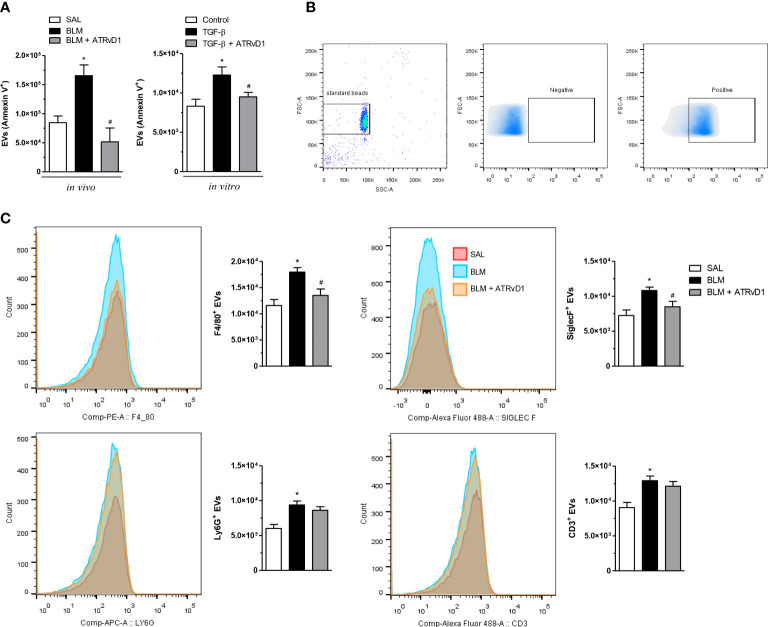
ATRvD1 inhibits the release of extracellular vesicles (EVs) *in vivo* and *in vitro*. **(A)** EVs were isolated from the BAL obtained on day 14 after the SAL, BLM, and BLM + ATRvD1 treatments or from the conditioned medium harvested 24 h after the macrophages were incubated with TGF-β (10 ng/ml) or TGF-β + ATRvD1 (100 ng/ml). The EVs were stained for FITC-labeled annexin V and analyzed by flow cytometry. **(B)** Dot-plots and density-plot images show the gate strategy for EVs: **(i)** the gate region of large vesicles using 1-μm standard beads (left); **(ii, iii)** gates were defined from unlabeled vesicles (negative), being considered positive for the specific markers of the fluorescence captured following that point. **(C)** The EVs obtained from BAL were also stained for F4/80-PE (macrophages), SinglecF-alexa488 (eosinophils), Ly6G-APC (neutrophils), and CD3-alexa488 (lymphocytes) and analyzed by flow cytometry, and fluorescence was plotted as histogram and bar graphs of absolute number. Results represent mean + SEM from three independent experiments. *P ≤ 0.05 vs. the SAL/control; ^#^ *P* ≤ 0.05 vs. the treated groups (BLM or TGF-β).

We evaluated the influence of ATRvD1 treatment on the production and release of EVs. BLM injection duplicated the EV contents in BAL compared with control mice. Interestingly, ATRvD1 administration impaired BLM-induced EV release, restoring the EV numbers to those observed in the BAL of SAL-treated mice (**Figure 4A**, left panel). In addition, using an *in-vitro* strategy, in which macrophages were cultured with or without the presence of TGF-β, we also tested the ATRvD1 effect. As noted in **Figure 4A** (right panel), macrophage incubation with TGF-β caused an increase in the number of EVs released to the conditioned medium compared with the supernatants from untreated cells, while ATRvD1 incubation impaired TGF-β-induced EV production.

Additionally, in an attempt to determine the cellular source of the EVs, we first used 1-μm standard beads to define the gate region strategy as shown in panel B of **Figure 4**. We observed that BLM induced an increase in F4/80^+^, SiglecF^+^, Ly6G^+^, and CD3^+^ EVs recovered from BAL fluids. Remarkably, ATRvD1 treatment decreased just F4/80^+^ and SiglecF^+^ EV release but did not modify the event number of Ly6G^+^ and CD3^+^ EVs (**Figure 4C**). Our results uncovered a new mechanism exerted by ATRvD1 in macrophages, as an important modulator of EV formation/release.

### ATRvD1 inhibits BLM-induced angiogenesis

**Figure 5:**
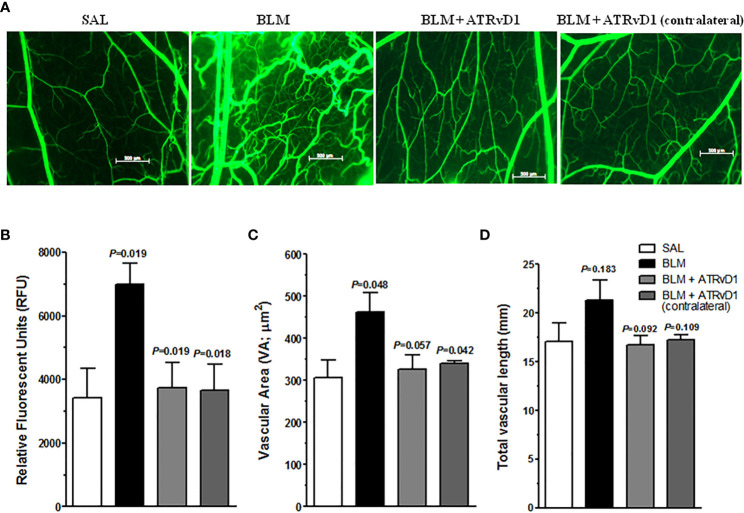
ATRvD1 treatment inhibits BLM-induced angiogenesis in HCP. **(A)** Representative fluorescent light images of HCP preparation after SAL, BLM (1 U/100 µL/hamster – injected into the cheek pouch), BLM + ATRvD1 (25 µg/kg - 100 µL i.v.) treatments, and contralateral images of HCP in BLM-treated hamsters. The image area is 5 mm^2^. **(B–D)** Microvascular alterations were quantified by measuring the fluorescence measurement (RFU), vascular area (VA), and total vascular length (TVL). Results represent mean ± SD from independent experiments. The p-value on the top of the BLM bar represents the comparison to the SAL group, while the others values were compared to the BLM group.

Evidence in the literature demonstrates that angiogenesis is a crucial step in fibrosis development (32). Moreover, it is also demonstrated that RvD1 presents an anti-angiogenesis effect in a model of corneal neovascularization (33). Using the intravital fluorescence microscopy technique in the HCP model, we observed that BLM administration *in loco* promoted an intense angiogenesis evaluated at 24 h by FITC-dextran (fluorescent tracer), compared with SAL administration (**Figure 5A**). ATRvD1 treatment prevented BLM-induced neovascularization (**Figure 5A**). These data were quantified by the total fluorescence measurement as shown in **Figure 5B**. The contralateral cheek pouch in the BLM-induced hamster did not present angiogenesis (**Figure 5A**). Our data demonstrated that ATRvD1 seems to prevent angiogenesis, contributing to the antifibrotic effect of BLM.

Several parameters can be obtained from the intravital microscopy technique (31, 34). Among them, we analyzed the vascular area and the total vascular length. BLM induced a considerable increase in the vascular area compared with the values observed for the SAL-treated HCP or in the contralateral BLM-treated HCP (**Figure 5C**). Meanwhile, ATRvD1 treatment recovered the BLM effect (**Figure 5C**). Similarly, when we analyzed the total vascular lenght, we also observed that BLM induced an increase in total vascular length compared with SAL-treated animals, which was inhibited by ATRvD1 treatment, however it did not reach statistical difference (**Figure 5D**).

## Discussion

SPMs are important mediators during the resolution phase of the inflammatory process (35). In the present study, we explored ATRvD1 as a possible treatment for lung fibrosis in a BLM-induced murine model. In fact, the same question was previously addressed by Yatomi and colleagues, which reported that ATRvD1 treatment reduced lung cellularity, IL-1β and TGF-β mRNA expression, and matrix protein (collagen I) deposition and rescued lung morphology (21). Nevertheless, our study not only corroborates with the literature but also considers new ATRvD1 underestimate pro-resolution mechanisms. Here, we confirmed these observations while additionally showing that ATRvD1 reverts fibroblast differentiation and inhibits the release of EVs *in vivo* and *in vitro*, reduces fibronectin deposition, and abolishes angiogenesis in HCP tissues challenged by BLM.

Pulmonary fibrosis is characterized by abnormal wound healing, leading to matrix protein accumulation with loss of elasticity and consequent organ dysfunction. The mechanisms involved in these fibrotic processes are intricate and not fully understood; however, it is well known that fibroblast activation, proliferation, and differentiation, in association with imbalanced immune and inflammatory responses, underlie pathogenic effects. Given the precedent in which the other authors observed a reduction in neutrophil and monocyte recruitment into the lungs of fibrotic (21) and infected (36) mice treated with ATRvD1, it was predictable that ATRvD1 treatment would likewise limit leukocyte infiltration and activation in the lung of BLM-treated mice. Indeed, Schmid and colleagues demonstrated that the antifibrotic effect of ATRvD1 was partly explained by inducing macrophage polarization toward M2 non-fibrogenic phenotype, in addition to reducing the secretion of IL-1β and IL-8; abolishing chemotaxis to chemerin, formyl-methionyl-leucyl-phenylalanine (fMLP), and macrophage chemotactic protein-1 (MCP-1); and enhancing the phagocytic activity of microbial particles (37). In the present study, we observed an increase in CD86^+^ (M1) and CD206^+^ (M2) cells in the lung and BAL of BLM-injected mice, in which ATRvD1 treatment partly reduced CD86^+^ and CD206^+^ interstitial macrophages (lung), showing no effect on alveolar macrophages (BAL). Our data demonstrated a possible role of ATRvD1 in attenuating M1- and M2-polarized macrophage populations in the lung, indicating a non-preferable cell activation but a reduction in inflammatory profile. It is also important to have in mind that such an effect on macrophages could be mediated by an indirect mechanism *in vivo*, and different experimental protocols should be applied to explore this aspect.

The finding that ATRvD1 may limit the progression of pulmonary fibrosis or even reverse an established fibrotic process is supported by studies in different mouse models, such as BLM-induced lung fibrosis (21), stretch-induced lung fibrosis (17), and unilateral ureteric obstruction-induced kidney fibrosis (38). Using a combination of experimental approaches, our study reinforces the concept that ATRvD1 can restore lung morphology by regulating the production and deposition of structural components such as extracellular matrix proteins *via* the regulation of MMP-9. Indeed, in our study, MMP-9 and TIMP-1 expressions were induced by BLM, but only MMP-9 was inhibited by ATRvD1. In contrast, Yatomi and colleagues (21) reported that MMP-9 expression was inhibited by BLM and reverted by ATRvD1, while no difference was observed for TIMP-1 expression. The discrepancy in MMP-9 expression is probably due to continuous BLM administration *via* a subcutaneous micro-osmotic pump used in the study of Yatomi, which confers a systemic effect of BLM. Also, MMP-9 detection was evaluated by mRNA expression, while we detected protein expression.

In addition, here, we found that ATRvD1 dramatically reversed the inflammatory/fibrotic environment induced by BLM. The reduction of TGF-β levels, together with other cytokines, after ATRvD1 treatment, may have an impact on fibroblast activation and differentiation. Substantially, ATRvD1 impaired fibroblast differentiation in *in-vitro* and *in-vivo* models, and such effects could justify the mitigation of exacerbated tissue remodeling, promoting lung architecture preservation. Despite the impressive impact of ATRvD1 treatment, it is unclear to what extent these effects contribute to the antifibrotic activity of this SPM.

There is a large body of evidence describing the inflammatory process induced by BLM (i.t.) lasts up to 5 to 7 days, where fibrosis markers are already present. So, this time point would be interesting to begin antifibrotic treatment, as recently revised by Kolb et al. (39). In another study, on day 7 post-BLM challenge, the lung tissue displayed inflammatory cell infiltration, vascular congestion, thickened septa, alveolar collapse, and increased Masson’s trichrome staining (40). Similar results were also observed using rats in a BLM-induced fibrosis model, in which a significant collagen deposition was also noted 7 days post-BLM administration (41). Substantially, we also detected collagen deposition, cellular infiltration (**Supplementary Figure 1**), and reduced pulmonary function, evaluated by mechanical ventilation (23), on day 7 after 0.06 U/mouse of BLM. Taken together, we are confident that ATRvD1 treatment on day 7 reverts the ongoing fibrotic process in the lung.

Especially in the past decade, a large body of evidence has emerged pointing to EVs as an important mechanism of intercellular communication. EVs were first identified by Peter Wolf in 1967, determined initially as cellular trash during research on coagulation, and termed as platelet dust [cited in (42)]. Nowadays, EVs are well-recognized for their association with several diseases such as atherosclerosis, diabetes, cancer, sepsis, pulmonary hypertension, cystic fibrosis, and idiopathic lung fibrosis (42–44). It was recently published that polymorphonuclear leukocyte-derived exosomes are found in chronic obstructive pulmonary disease and modulate matrix destruction by proteolytic damage (45). Still, there is evidence of increased levels of exosomes with profibrotic characteristics in the circulation of patients with IPF, as well as in the serum of murine bleomycin-induced lung fibrosis (46). Our data also demonstrated an upregulation of EVs in the BAL obtained from BLM-injected mice, derived from macrophages, neutrophils, eosinophils, and T cells. Using TGF-β-stimulated cultured macrophages, we confirmed the release of EVs. Notably, ATRvD1 treatment impaired the release of EVs both *in vivo* and *in vitro.* Although we have not investigated the role and contents of EVs, our data corroborate to the pro-inflammatory and profibrotic effect of EVs released after bleomycin insults. It remains to be determined whether ATRvD1 impairs the formation, the cargo content, or the release of EVs. Further studies are needed to bring insights into the function and contents of EVs in BLM-induced fibrosis and if ATRvD1 regulates such processes.

The influence of angiogenesis in pulmonary fibrosis was first described by Turner-Warwick in 1963 (47). Data in the literature suggest that neovascularization supports the fibrosis process, thereby allowing fibroblast proliferation and extracellular matrix deposition (48). The role of angiogenesis in fibrosis development was confirmed using an anti-angiogenic peptide which conferred protection against BLM-induced pulmonary fibrosis (49). Here, ATRvD1 reduced BLM-induced angiogenesis in the hamster cheek pouch model. Owing to technical limitations (intravital microscopy), these sets of experiments were performed in hamsters, not in mice. We show here for the first time that ATRvD1 has anti-angiogenic effects in the hamster microcirculation; however, we still have to determine if ATRvD1 is also able to inhibit BLM-induced angiogenesis in mice. Noteworthy, it was previously reported that lipoxin A_4_ inhibits endothelial cell proliferation and migration (50), raising the possibility that ATRvD1 could also prevent angiogenesis by inhibiting endothelial cell remodeling. However, this hypothesis remains to be further explored, considering conflicting data showing that ATRvD1 treatment enhances vascular remodeling in a different model of sterile inflammation (51). Further studies are necessary to elucidate the mechanisms behind ATRvD1 inhibition on BLM-induced angiogenesis.

Resolvins are characterized as SPMs produced during the inflammatory response. Although the most well-studied SPM is lipoxins, particularly lipoxin A_4_, which share many characteristics with resolvins, including anti-inflammatory and pro-resolution actions, both were mediated by the same receptors, ALX/FPR2 (expressed in mouse and human) and GPR32 (expressed only in human) (52). We have already determined the expression of ALX in our pulmonary fibrosis model previously (53). At that time, we did not detect any difference in ALX expression in the whole lung obtained 21 days after the BLM challenge compared with saline-treated mice. In the current study, we also determined the message for ALX by PCR in the lungs harvested 14 days after the BLM challenge for all the groups. Unlike what was observed in our previous work, ALX expression was reduced in the lungs obtained both from BLM and ATRvD1-treated BLM samples, compared with the SAL group. On the other hand, the ALX mRNA expression induced by TGF-β in primary murine fibroblast did not show significant differences among the groups. In the current work, it is important to emphasize that we detected mRNA expression evaluated on day 14 in BLM-induced fibrosis or after 24 h of TGF-β stimulus in primary culture cells. In our previous work, we evaluated the receptor protein ALX on day 21. Even so, we think that the BLM putative impact on ALX mRNA expression probably did not interfere with the ATRvD1 effects observed in the BLM-induced fibrosis, since it reverted practically all the parameters analyzed.

The pharmacological therapy for pulmonary fibrosis is currently based on corticosteroids and antifibrotic and immunosuppressive agents, which are not always effective. Furthermore, several other therapeutic molecules and pathways with potent antifibrotic effects were described in the past decade, such as metformin, tumor necrosis factor-related apoptosis-inducing ligand (TRAIL), pentraxin 3 (PTX3), protein kinase Cζ and δ, angiotensin/AT2, maresin 1, and aspirin-triggered lipoxin A4 (ATLA/FPR2), which bring new perspectives to the development of an appropriate pharmacological treatment for pulmonary fibrosis (53–60).

In summary, the present study shows that ATRvD1 exerts a therapeutic effect on BLM-induced lung fibrosis, using a combination of *in-vivo* and *in-vitro* models. Our work suggests that ATRvD1, presenting antifibrotic effects, needs to be considered for therapeutic purposes. These results reinforce and encourage the scientific and medical society to consider SPMs as new therapeutic agents for lung fibrosis.

## Data availability statement

The raw data supporting the conclusions of this article will be made available by the authors, without undue reservation.

## Ethics statement

The animal study was reviewed and approved by the CEUA—Comite de ética de uso animal da UFRJ.

## Author contributions

CC and CB conceived the idea and project, wrote, and edited the manuscript, created the graphs, and revised the manuscript. RG, JS, TN, IW, VF and CP performed the experiments and analyzed the data. CB and PB assisted with the microvascular computational analysis. RS and ES performed the intravital microscopy experiments, edited the manuscript, and analyzed the data. JM assisted with the microparticle experiments and data collection. NM, CS and FL performed immune-staining assay and analysis. JS edited the manuscript and discussed the results. All authors contributed to the article and approved the submitted version.

## Supplementary material

Supplementary Figure 1 Early fibrosis markers 7 days after bleomycin i.t. challenge. Lungs were harvested on the 7^th^ day after SAL or BLM (0.06 U/mouse in a final volume of 30 μL of SAL) challenges, as described in the Materials and Methods section. **(A)** Representative lung sections from SAL (left) and BLM (right) groups stained for hematoxylin and eosin. **(B)** Picrosirius red staining under bright field (superior images) and polarized light (lower images) images obtained from the same groups. Pictures are representative of each group, constituted of n=4. Bar scale = 50 μm.
