# Supplementary figures and images for: Pleiotropic antifibrotic actions of aspirin-triggered resolvin D1 in the lungs
Source: Front Immunol. 2023 Mar 7;14:886601. doi: 10.3389/fimmu.2023.886601 (PMC10030054; doi:10.3389/fimmu.2023.886601)

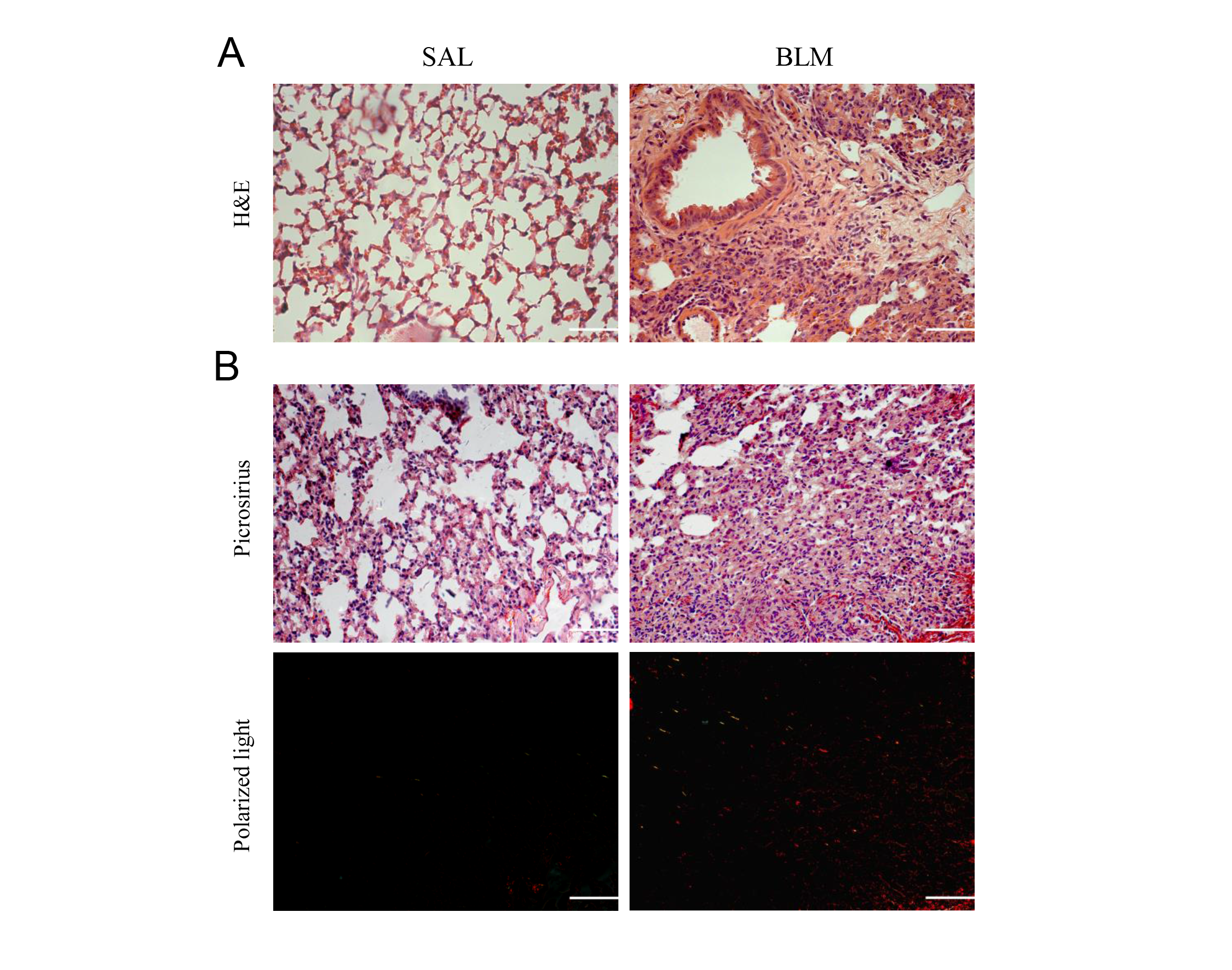

Supplement: Supplementary Figure 1 — Early fibrosis markers 7 days after bleomycin i.t. challenge. Lungs were harvested on the 7th day after SAL or BLM (0.06 U/mouse in a final volume of 30 μL of SAL) challenges, as described in the Materials and Methods section. (A) Representative lung sections from SAL (left) and BLM (right) groups stained for hematoxylin and eosin. (B) Picrosirius red staining under bright field (superior images) and polarized light (lower images) images obtained from the same groups. Pictures are representative of each group, constituted of n=4. Bar scale = 50 μm. [file Image_1.tif]
